# Young Muslim Women Living with Asthma in Denmark: A Link between Religion and Self-Efficacy

**DOI:** 10.3390/pharmacy6030073

**Published:** 2018-07-23

**Authors:** Louise C. Druedahl, Duaa Yaqub, Lotte Stig Nørgaard, Maria Kristiansen, Lourdes Cantarero-Arévalo

**Affiliations:** 1 Social and Clinical Pharmacy Group, Department of Pharmacy, Faculty of Health and Medical Sciences, University of Copenhagen, Universitetsparken 2, DK-2100 Copenhagen, Denmark; iwear_aps@hotmail.com (D.Y.); lotte.norgaard@sund.ku.dk (L.S.N.); lou.cantarero@sund.ku.dk(L.C.-A.); 2 Department of Public Health, Faculty of Health and Medical Sciences, University of Copenhagen, Øster Farimagsgade 5, DK-1014 Copenhagen, Denmark; makk@sund.ku.dk

**Keywords:** adherence, asthma, Bandura, Denmark, ethnic minority, self-efficacy, young adults, women

## Abstract

Asthma is a chronic respiratory disease that can be controlled with appropriate medicinal treatment. Adherence to pharmacological treatment is therefore critical. Self-efficacy plays a key role in adherence to medicine in chronic diseases, including asthma. Additionally, ethnic minorities have poor adherence to medicines. However, the impact of religion on self-efficacy and adherence is understudied. Therefore, the aim of this study was to explore the role of self-efficacy in adherence to asthma medicine treatment and the influence of religion on self-efficacy among young, Muslim minority women. A focus group and individual interviews with 10 Muslim minority women (14–24 years of age) living in Denmark were conducted. Data analysis was deductive using Bandura’s theory of self-efficacy and modes of agency. Overall, religion was shown to affect self-efficacy. The women reported changes in self-perceived self-efficacy during the holy month of Ramadan. In addition, praying was used as an alternative to medicine for controlling asthma symptoms. However, the women did not perceive religion and treating asthma with medicine as mutually exclusive, but rather as coexisting for the shared goal of controlling asthma symptoms. It is important for healthcare professionals (HCPs) to be aware of the link between self-efficacy, religion and adherence to asthma medicine treatment. This awareness can aid HCPs in giving advice regarding adherence to asthma treatment, and when monitoring treatment to improve the quality of asthma care for young Muslim minority women.

## 1. Introduction

Asthma is a chronic inflammatory airway disease that affects people irrespective of their gender, age and country of birth [1,2,3]. Asthma is the most common chronic disease affecting the lower respiratory tract in children [3]. More boys than girls are affected by asthma, although by young adulthood, this pattern either cannot be detected or has reversed [2,4]. Hormonal changes are thought to contribute to the shift in gender-dependent disease patterns [4,5]. In 2017, the World Health Organization estimated that about 235 million people worldwide have asthma [6], and that the prevalence is increasing [7].

Minimising the health impact of asthma requires individuals to have the necessary skills to manage their disease. Most patients can manage the symptoms successfully using pharmacotherapy, patient education, symptom monitoring and/or by avoiding triggers [3]. Treatment includes long-term control and fast-acting relief medicine, the former to prevent symptoms and the latter for acute symptom relief [3,8,9]. Correct use of controller medicine such as inhaled corticosteroids (ICS) can control asthma symptoms and reduce the risk of exacerbation [8]. However, adherence to ICS treatment is low: 49–73% for children and less than 50% for adolescents and adults [10]. One reason for the low medicine adherence of adolescents could be that healthcare professionals (HCPs) do not provide sufficient preparation or support to parents and/or affected children for the transition in responsibility for medicine [11]. Parents or caregivers are responsible for managing asthma in children, including redeeming prescriptions, communicating with HCPs and administering medicine [11]. For the future adult to take on this responsibility, the transfer must be successfully made starting in the early stages of adolescence [11]. One relevant aspect for this is self-efficacy, which is an individual’s belief in his/her own ability to perform a specific action to manage a particular situation [12,13]. Furthermore, self-efficacy is essential for managing asthma [14,15,16] and for medication adherence in general [17].

Ethnicity is a complex concept that describes the group to which persons belong/are perceived to belong on basis of shared characteristics such as geographical, language and cultural traditions, but may also include facets of religion [18]. Some ethnic minority patients have a higher disease burden of asthma than ethnic majority patients, illustrated by their increased rate of hospital admissions compared with the majority population [19,20]. In Scotland, the highest hospital admission rate was seen among Pakistanis [20]. Ethnic minority also seems relevant for adherence to controller asthma medicine. Studies in the Netherlands and USA, respectively, have found that ethnic minority children and adolescents have the same [21] or lower [22] adherence to controller asthma medicine than the majority population. A study in UK found children of parents from Pakistan to more often have wheezing the last 12 months, but also physician’s diagnosis of asthma and medicine usage compared to British background [23]. Although asthma morbidity and mortality have been found disproportionately high in ethnic minority children, cultural tailoring of treatment is rarely incorporated into treatment regimens [24]. A Danish study comparing Danish ethnicity to Turkish, Lebanese and Iraqi ethnicity found that ethnic inequalities in asthma treatment were not caused by differences in household income, but may result from a lack of parental knowledge about the Danish healthcare system [25]. Other factors affecting ethnic inequalities in asthma include poor health literacy, religious and health beliefs and challenges in cross-cultural communication [26]. One example hereof is that Muslims may prefer to avoid using medicine during Ramadan [27]. However, overall, supported self-management from HCPs can possibly improve quality of life for ethnic minority patients with asthma [28].

In Denmark, asthma prevalence is higher among boys than girls, but with a shift in proportion in adolescents and young adults [29]. In addition, women with asthma (above 16 years of age) have more emergency department visits and a higher societal disease burden compared with men [29]. Therefore, the aim of this study was to explore self-efficacy to adhere to asthma medicine treatment to assess the influence of religion on self-efficacy and adherence among young, ethnic minority women ages 14–24.

### 1.1. The Setting

In Denmark in 2015, non-Danish origin comprised 11.7% of the population [30]. Most of these were first generation immigrants (8.9%), and the remainder were their descendants (second generation 2.8%) [30]. The largest groups originated from non-Western countries: Turkey, Iraq, Pakistan, Bosnia-Herzegovina, Iran and Lebanon [30]. The definitions of ‘immigrant’ and ‘descendant’ are based on definitions of Statistics Denmark [30]. According to these, ‘immigrants’ are born outside of Denmark and none of the parents are both born in Denmark and a Danish citizen. Furthermore, ‘descendants’ are born in Denmark, but none of the parents are both born in Denmark and a Danish citizen. In addition, ‘Danish origin’ is if one of the parents is both born in Denmark and is a Danish citizen [30].

Ethnic minority is in Denmark defined by the country of origin of the parents. Middle Eastern countries comprise five out of six of the largest non-Western groups in Denmark and all where Islam is the official religion, and therefore interviewees with Middle Eastern origin practicing Islam was the focus of the study.

### 1.2. Theory of Self-Efficacy

As previously mentioned, Albert Bandura’s theory of self-efficacy describes an individual’s belief in his/her own ability to perform a specific action to manage a particular situation [12,13]. The theory relates to behavioural choices, which may or may not have adverse effects [12]. Therefore, self-efficacy can be used for the health-related behaviour of taking asthma medicine.

Self-efficacy is linked to the individual’s expectations to reach a certain outcome. Achieving the outcome is influenced by the individual’s expectation about (i) his/her ability to behave in a certain way, and (ii) the behaviour resulting in the desired outcome [12]. A person with high self-efficacy will increase his/her efforts when faced with difficulties. In contrast, a person with low self-efficacy may behave ineffectively despite knowing what to do [13].

Although the theory of self-efficacy does not address religion [12], Bandura briefly links religion to self-efficacy via the concept of agency [31]. Three modes of agency are relevant to religious practice: personal agency, proxy agency and collective agency [31]. For example, personal agency increases if religious teachings inspire an individual to believe that he/she can exert control over his/her own actions and surroundings [32]. Active proxy agency relates to the belief that a divine or supreme being (usually referred to as God) can exercise power over events [31]. The effect of divine proxy agency can be either to increase self-efficacy, if God is perceived as a collaborating partner, or to cause passivity (i.e., reduce self-efficacy), if God is being depended on to solve problems experienced [31]. Prayers are a way of invoking proxy agency, which individuals often use in times of distress due to physical or emotional problems [31]. Collective agency refers to the pooling of knowledge, resources and skills, and builds on the perception of people as social (not isolated) creatures [31]. Religious beliefs can provide both psychological support and a model for behaviour [31].

Lower self-efficacy may be associated with an ethnic minority background [33], and with more emergency department visits and hospital admissions [34]. Patients with asthma who have high self-efficacy link to a higher quality of life [35], fewer asthma symptom days [33] and higher adherence to controller medicine [36]. Moreover, high adherence to asthma controller medicine use links with positive outcome expectations, which influences self-efficacy [36]. A recent study found that self-efficacy was more important to asthma outcome than knowledge about asthma, attitudes towards asthma management and perceived barriers to care [34]. Thus, improvement in self-efficacy among asthma patients may lead to better self-management and disease control [14,37,38].

## 2. Materials and Methods

A qualitative approach was used. Data were collected between May and July 2016, via a focus group and individual interviews with young, Muslim minority women. The data were collected in the capital region of Denmark.

### 2.1. Recruitment

Convenience sampling via Facebook was used to recruit 14–24-year-old Muslim minority women who had asthma and treated asthma with medicine. Other inclusion criteria include Middle Eastern origin (see Section 1.1) and practicing Islam. The presence of asthma was self-reported by interviewees. The first 10 eligible interviewees were selected. The recruitment included posting several calls for interviewees in 11 Facebook groups or pages for ethnic minority groups in Denmark. The language of the calls was Danish. The calls were posted by the second author, a young, bilingual Danish-Arabic female pharmacist who shares ethnic and religious background with the interviewees.

### 2.2. Conducting Interviews

The study sample comprised 10 interviewees, all of whom participated in an individual interview and were invited to participate in a focus group. All 10 individual interviews and the single-focus group interview of three interviewees were semi-structured. The second author constructed the interview guides, which were informed by a systematic review of literature in the PubMed and Embase databases on ethnic and minority groups, self-efficacy and anti-asthmatic agents. The interview guide used for individual interviews was designed to encourage interviewees to describe their experience of everyday life with asthma medicine, including their use of opinions and attitudes about asthma medicines, as well as coping strategies for handling exacerbations. Thematic content in the interview guide for individual interviews was asthma history, medication compliance, social aspects of having asthma, ethnicity, religion, limitations due to asthma and self-efficacy. Themes for the focus group were medication compliance, ethnicity and the Muslim holy month of Ramadan. See the interview guides in Supplementary Material. To explore the influence of religion on medication adherence, questions addressing the influence of faith and religious practice on the course of the disease and medicine use were included in both of the interview guides.

The second author conducted and moderated all interviews in Danish. Individual interviews were carried out either in person or by online video call, according to the interviewee’s preference. The focus group interview was conducted in person at the University of Copenhagen. Each interviewee was given one gift certificate of 100 DKK (~13 EUR or ~16 USD) for participating. All interviews were audio-recorded and transcribed verbatim. Quoted text from interview transcripts was translated from Danish to English by the first author. The interview guides were translated from Danish to English by the first and third authors.

### 2.3. Data Analysis

The data were deductively analysed using Bandura’s theory of self-efficacy [12,13,31,39] and three types of agency relevant to religion [31] to explore the experiences of young, Muslim minority women with self-efficacy and adherence to asthma medicine treatment.

De-identified interview transcripts were analysed by extracting all text with behavioural, emotional or belief-related content on relevant themes. The themes were classified into low and high categories as part of the process. The themes were:

• Outcome expectations (high/low)

Expectations were labelled high outcome, if the interviewee indicated that using medicine would provide higher control of asthma symptoms. Similarly, expectations were labelled low outcome, if the interviewee described using medicine as inadequate to improve control of asthma symptoms.

• Self-perceived self-efficacy to use asthma medicine (high/low)

Self-perceived self-efficacy was the interviewee’s perception of her ability to use asthma medicine. If this was not described, the interviewee’s general self-efficacy was assessed instead based on general statements about asthma-related experiences and whether the interviewee described having a more positive or more negative attitude (corresponding to high or low general self-efficacy, respectively).

• Adherence to asthma medicine treatment (high/low)

Adherence (to asthma medicine) was assessed based on the interviewee’s statements about actual medicine use

Interview transcripts were de-identified prior to analysis by the second author. Data were analysed by the first author, who is also a pharmacist, but does not share a cultural or religious background with the interviewees. All co-authors discussed the analysis to ensure rigor and that the analysis reflected the data as a whole.

### 2.4. Ethics

According to Danish [Order no. 410 of 09/05/2012] [40], ethics approval is not necessary when personal data are handled as part of a master’s thesis research project, as long as the interviewee provides explicit consent. According to the Danish Data Protection Agency, these personal data include data obtained from interviews. The interview data in this study were handled as part of the second author’s master’s thesis research project. Written informed consent to participate in the study was obtained from all interviewees or parents of minors (age < 18 years) on behalf of their child.

## 3. Results

The study sample comprised 10 interviewees with self-reported asthma, who identified themselves as Muslims. Interviewees were 14–24 years old and had had asthma for 2–18 years. All interviewees used inhaler asthma medicine. All interviewees participated in individual interviews and three (IP3, IP6 and IP8) also participated in a focus group interview held after the individual interviews. Individual interviews varied in length from 30 min to one hour, and the focus group interview lasted for one hour. Demographics of the interviewees can be seen in Table 1.

### 3.1. Perceptions of Asthma and the Experience of Having Asthma

All interviewees said that they tried to live a normal life despite their asthma and its treatment. They stated that they spend a lot of time with their friends and using asthma medicine is not an issue with their friends, and that they do not feel stigmatised by having the disease. Even so, the experience of using asthma medicine varied among interviewees. For example, IP1 (15 years old) described an episode in which she experienced relief after using fast-acting reliever medicine to control her asthma symptoms. However, IP6 (21 years old) described a contrasting episode in which she took reliever medicine just prior to her annual or semi-annual hospitalisations, but did not achieve the expected symptom relief: “*If I usually use [terbutaline, relief medicine] and it doesn’t help, it gets even worse*” *(IP6).* Observing relatives with asthma using asthma medicine seemed to reassure the interviewees by helping them to contextualise their situation as normal and manageable. However, the way the interviewees thought about their illness differed profoundly: five saw asthma as unproblematic or not chronic, while others did perceive asthma as a problem and believed it would continue to be so throughout their lifetime. For example, IP10 stated that she can remain calm when having an exacerbation and that she can get rid of asthma, whereas IP9 said she is nervous about having to live with asthma for the rest of her life. Remaining calm or nervous during an asthma attack were unrelated to the interviewees’ age or age of onset of asthma.

Focus group interviewees distinguished their ethnicity and described their feelings of being ethnic minority patients due to traditions, culture and religion. A re-occurring theme in both individual and focus group interviews was that ethnicity and religion influence self-efficacy, which will be elaborated on in the following paragraphs.

While some interviewees claimed high self-efficacy in their approach to asthma medicine, their self-described behaviour mainly comprised examples of not using the medicine (i.e., low self-efficacy; Table 2, see e.g., IP7). Please see Table 2 for an overview of the adherence of each interviewee with regard to her medicine use, her expectations of how medicines will affect her asthma symptoms and her assessed level of self-efficacy.

### 3.2. Expectations about Medicine to Control Asthma Symptoms

Interviewees used other ways to control their asthma symptoms besides asthma medicine, which relates to Bandura’s different modes of agency related to religion.

Despite technical competency in using inhalers, interviewees indicated a lack of knowledge about the outcome from using asthma medicine (especially long-term controllers). For example, IP9 did not consider it necessary to remind herself to take her medicine, because she knew her cough would remind her, which means that she failed to effectively adhere to controllers to prevent asthma symptoms: “*I don’t have to do anything [to remember to use medicine]. When I don’t use it, my coughing reminds me right away*” *(IP9)*. Therefore, not all interviewees linked the action of using medicine to the outcome of controlling asthma symptoms. In addition, a few interviewees said that they became better at remembering to use the controller medicine when they experienced more frequent asthma symptoms.

Some interviewees had low expectations about the controller medicine as a means to prevent asthma symptoms. For example, IP1, IP3, IP4, IP5, IP7 and IP9 stated that long-term control medicine was unable to improve their sense of well-being because it lacked an immediate effect (unlike fast-acting reliever medicine). For this reason, controller medicine was believed to have less effect than reliever medicine (IP6, IP7 and IP9). However, some interviewees indicated that the controller medicine had an effect. For example, IP6 said that she knew the controller medicine helped without having a perceptive effect: “*The brown [controller medicine]/…/ does not [seem to] help, but it helps without my feeling it*” *(IP6)*.

### 3.3. Influence of Religion, Modes of Agency and Ramadan on Self-Efficacy and Asthma Medicine Adherence

Religion was crucial to the interviewees in managing their asthma. All interviewees described religious practices such as praying or fasting during Ramadan. Interviewees described religion as influencing their feelings about having and managing asthma. For IP9, this meant that the effect of reading a religious text, a hadith, helped her to feel more positive and strengthened her belief in her abilities to manage her asthma. “*Without my religion, I don’t think I would have seen any good sides of the illness/…/It [asthma] heavily affects my everyday life/…/but according to my religion, Allah Teala [God] gives an illness to someone to cleanse their sins/…/I see it [asthma] as an opportunity to cleanse my sins/…/He [God] loves me so much that he wants me in Paradise, so I have been blessed with an illness/…/and hadith [religious text]/…/has motivated me and given me a positive approach*” *(IP9)*. This example related to active proxy agency as well as personal agency, but it was the only example of personal agency described by an interviewee.

The most frequently expressed mode of agency was active proxy agency: most interviewees (seven out of 10) described the supreme being (referred to as God or Allah) as an active proxy agent. This was illustrated, for example, by praying for help from Allah to be cured of asthma or to increase self-efficacy to use asthma medicine. “*I ask Allah if he could please make my asthma better*” *(IP8).* Some interviewees considered an improvement in asthma symptoms to be an answer to prayers or a reason to be thankful to Allah (IP4, IP6, IP7 and IP8). The interviewees described Allah as exercising power over events and said that Allah ensured control of their asthma symptoms. Overall, achieving control of asthma symptoms was described as the result of using medicine as well as the answer to prayers.

However, praying was also described as a collective agency; for example, IP8’s mother also prayed for improvement of her daughter’s asthma: “*And my mom, she also always asks Allah to make me healthy in her prayers*” *(IP8).*

The interviewees described fasting during the holy month of Ramadan as an essential part of their religious practices. Opinions among the interviewees appear to differ about whether or not taking medicine means breaking the fast. IP3 stated that it was acceptable not to fast when having an illness even though she did not want to take medicine when fasting, whereas IP7 and IP9 expressed the opinion that taking medicine does not break the fast. However, IP5 was not sure whether it was *haram* (sinful) to use medicine during Ramadan if she felt ill from asthma, and IP3 was determined not to break her fast despite experiencing asthma symptoms that previously had led to a two-day hospitalisation. Interviewees sought information about whether or not it was considered appropriate to use medicine during Ramadan from their families, the Internet and religious texts. Interviewees differed as to whether they included physicians in their decision-making about using asthma medicine during Ramadan. IP2 and IP8 said that they discussed the new treatment regimen with their physicians, whereas IP6 and IP9 made decisions without their physician’s advice.

All interviewees wanted to fast, but their abilities to do so differed. IP8 fasted for a couple of days even though her physician told her not to, so that she could take her medicine, whereas IP6 stated that she was unable to fast for seven days because of her asthma. The focus group interviewees indicated that they were unaffected by verbal attempts at persuasion from parents, husbands and physicians to abstain from fasting.

All interviewees explained that they perceived to combine fasting with using medicine. Although some interviewees mentioned altering the dosage regimen, this was perceived by them as combining fasting with medicine use. Thus, fasting affected the self-perceived self-efficacy to use medicine. Most interviewees described aligning the intake of controller medicine with sunrise and sunset, for example: “*I will use it at suhoor [morning before sunrise] and at iftar [after sunset]*” *(IP5).* In contrast, IP3 and IP6 stated that using reliever medicine regularly as a safety precaution during Ramadan made them feel better psychologically even though they did not have an asthma attack when using it. The medicine regimen differed during Ramadan compared to other months of the year, although focus group interviewees emphasised that both fasting and asthma medicine use are important:

*IP3:* “*You should prioritise both [fasting and using medicine.]*”

*IP6:* “*… if you are only using the medicine once in the morning, then you shift it to somewhere in the evening. In that way, you are both fasting and complying with the medicine and thereby prioritising [them] equally high.*”

*IP3:* “*That way one still gets the same effect of the medicine…*”

*2nd author:* “*But is that something you also carry out? Rotating the time of using medicine depending on sunrise and sunset?*”

*All interviewees:* “*Yes*” *(focus group interview)*

Religion and religious practice played a large part in self-efficacy related to asthma health behaviour, including using medicine. According to the interviewees, using medicine and carrying out religious practices do not need to be mutually exclusive: the two can co-exist.

## 4. Discussion

The main finding of this study is that religion and religious practice influence self-efficacy to adhere to asthma medicine treatment.

The adherence to medicine treatment differed among these young, Muslim minority women. A reason for this might be found in their varying descriptions about the effect of their medicine. In particular, it was observed in this sample that low outcome expectations for controller medicine link to low adherence. This might be a part of the explanation for the reported lower redemption of prescriptions for asthma controller medicine compared to reliever medicine among Muslim minorities in Denmark [25]. This is supported by a study showing that adherence to asthma controller medicine links with positive outcome expectations [36]. Self-efficacy is a concept relevant for investigating asthma adherence [33,35,36]. Thus, it should be investigated whether low outcome expectations in general contribute to low adherence via its influence on self-efficacy.

### 4.1. Religion and Religious Practice Influence Self-Efficacy 

Medicine adherence for these young, Muslim minority women shifts during Ramadan, often to either once a day or twice a day, at both *suhoor* and *iftar*. Individuals with asthma have previously reported this pattern [27,41]. A review of the health impact of fasting during Ramadan recommended that individuals with asthma avoid fasting to prevent possible worsening of bronchoconstriction [42]. Similar to the findings by Azizi [42], this study reports that different traditions of Islam appear to provide different beliefs about whether inhaler use nullifies the fast, leading to inter-individual differences in adherence to asthma treatment during Ramadan compared with other months of the year.

All interviewees demonstrated religious commitment by their strong intention to fast despite their illness, which has also been reported for Turkish adults with asthma [41]. However, the interviewees generally expressed the opinion that they would break the fast if they felt ill, similar to Pakistanis with type 2 diabetes [43]. The self-efficacy to fast appears to be strong, despite verbal persuasion against fasting and the experience of worsening asthma symptoms as a consequence of fasting. This can be considered a counteractive influence on the self-efficacy to use asthma medicine.

Therefore, Bandura’s theory of self-efficacy [12,13,31,39] and modes of agency relevant to religion [31] cannot explain how religion shapes self-efficacy as revealed in this study.

The findings show that these young Muslim minority women with asthma perceive religion as offering an alternative (to using medicine) to obtain the desired outcome of controlling asthma symptoms. The interviewees used praying as part of religious practice to invoke the supreme being to exercise influence as an active proxy agent. A Danish study previously found that non-Western immigrants in Denmark may think their efforts to improve health are not important, which the authors report could be related to self-efficacy [44]. These results by Molin et al. [44] might be explained by the findings of this study connected with Bandura’s modes of agency. Bandura [35] states that passivity can develop due to a belief that God will help solve personal issues, and, in this present study, God was considered to be an active proxy agent in controlling asthma symptoms. Ahmedani et al. [45] reported that participants who believed that God determines health and health outcomes had lower ICS adherence compared to participants with other beliefs, although they did not explore the effects of a specific religion. A distinction can be made between a belief in God and a belief that God is an active proxy agent, and only the latter seems to influence asthma health behaviour. However, praying was also reported to heighten self-efficacy for using medicine, which is predicted to result in higher medicine adherence. 

It is important for HCPs to be aware of the link between self-efficacy, religion and adherence to asthma medicine treatment. An understanding of how these can interact can help both prescribers and pharmacy staff provide better counselling to young, Muslim minority women and aid the monitoring of treatment for this subgroup of patients. In turn, this could improve the quality of asthma care for young, Muslim minority women. 

### 4.2. Strengths and Limitations of the Study

The interviewer’s shared gender and religious background with the interviewees might have added to better interactions during the interview [46]. This also could have lead to muted opinions if thought to differ from the interviewer’s, but it was not the impression to be the case. Data saturation may not have been reached in this study due to the relatively small group of interviewees. Using the quality criteria of Lincoln and Guba [47], the authors sought to ensure the trustworthiness and transferability of data by transparency in data collection, data analysis and result reporting. The transferability is limited due to the small convenience sample, but may give an indication of possible opinions hold by young, Muslim women. One study strength was the reflexive discussion of data analysis among co-authors because this greatly strengthens the trustworthiness of results. 

The self-assessment of adherence can be a limitation of the study because other means were not used to ensure the validity of the assessment.

The recruitment strategy was successful in using social media, consistent with a previously conducted qualitative study that recruited participants in a comparable age range [48]. A potential issue is that this sampling approach does not target eligible participants not using Facebook. This might favour recruitment of interviewees who share the same views or beliefs, thus limiting data completeness. As it was not possible to discover why some potential recruits chose not to respond to the call for participation, it cannot be determined how responders differ from non-responders. However, the study did not aim to generalise the findings to a wider population, but instead aimed to gain insight into the experiences of young, Muslim minority women in Denmark using asthma medicine.

The theory of self-efficacy proved applicable for analysing adherence to asthma medicine treatment. However, it is important to be aware that dichotomisations, such as classifications of low and high self-efficacy levels in the present analysis, should be used with some caution. The insights into the lives of young, Muslim minority women obtained via the interviews are based on only a single time point in their lives; therefore, the self-perceived self-efficacy level might have shifted from low to high or vice versa if interviewees had chosen to tell other life stories. However, despite this limitation, it is of interest to understand the adherence and asthma health behaviour of this subgroup of patients.

### 4.3. Future Perspectives

More knowledge is needed about how to help Muslims combine fasting with adherence to asthma medicine treatment. A possible approach would be to explore the effect of providing health and medicine treatment information at mosques and by other means, as previously suggested for cancer treatment adherence in England [49]. In addition, there is a need for more research to update the theory of self-efficacy to conceptualise the impact of religion on self-efficacy.

## 5. Conclusions

Self-efficacy to adhere to asthma medicine treatment is linked to religion and religious practice. During the holy month of Ramadan, changes in self-perceived self-efficacy were reported by interviewees. In addition, praying was used as an alternative to medicine for controlling asthma symptoms. Feelings of improvement in asthma symptoms were considered as either an answer to prayers or a reason to be thankful to Allah. This was because the outcome of controlling asthma symptoms was perceived to be achieved by Allah. However, the use of medicine and religion was not perceived to be mutually exclusive, but rather to coexist with the shared goal of controlling asthma symptoms. 

It is important for HCPs to be aware of the link between self-efficacy, religion and adherence to asthma medicine treatment. The understanding of how these can interact can help both prescribers and pharmacy staff to provide better counselling to young Muslim women and aid the monitoring of treatment for this subgroup of patients. In turn, this could improve the quality of asthma care for young Muslim women.

## Figures and Tables

**Table 1:** Demographics of interviewees.

Pseudonym	Age	Years with Asthma	Medicine Prescribed ^1^ (Current Use)	Immigrant Status ^2^ (Country of Origin)
IP1	15	5	Reliever & controller (reliever only)	Descendant (Iraq)
IP2	14	2	Reliever & controller (both)	Descendant (Iraq)
IP3	19	16	Reliever & two types of controller (only reliever)	Immigrant (Iraq)
IP4	22	2	Reliever & controller (both)	Descendant (Palestine)
IP5	14	14	Reliever & controller (both)	Descendant (Palestine)
IP6	21	12	Reliever & controller (only reliever)	Descendant (Lebanon)
IP7	18	18	Reliever & controller (primarily reliever)	Descendant (Lebanon)
IP8	15	10	Reliever & two types of controller (all)	Descendant (Pakistan)
IP9	24	4	Reliever & controller (primarily reliever)	Descendant (Turkey)
IP10	20	10	Reliever & controller (both)	Descendant (Turkey)

^1^ Information about medicine use was obtained during interviews. ^2^ Descendant (born in Denmark) or immigrant was self-reported, and evaluated based on the definitions of Statistics Denmark [30].

**Table 2:** Overview of interviewees, their opinions, and the assessment of their self-perceived self-efficacy with regard to medicine use and their adherence to asthma medicine.

Pseudonym	Outcome Expectations (High/Low)	Self-Efficacy—General or in Relation to Medicine (High/Low)	Adherence to Asthma Medicine (High/Low)
IP1	High for reliever “*I use it [the medicine] and then I get tremors and after half an hour it works. And I begin to feel the effect*” Low for controller *[2nd author:* “*Do you think the controller medicine would help you not experience these asthma exacerbations?*”*]* “*No, it doesn’t matter*”	High (in general) “*[I am] an energetic young woman who fights for good results in life that can benefit me in my future*”	Low (for medicine) “*I tell her [the physician] that I don’t use my medicine*”
IP2	High “*Now I can feel when I need to use it/…/you don’t get better by not using it [the medicine]*”	High (for medicine) “*The blue [reliever] I always carry with me when I am not home, but I don’t use it that often. It is mostly the brown one [controller] that I use morning and evening every day*”	High (for medicine) “*In gym class when I felt ill, my teacher came and said that I should sit down /…/and use my medicine /…/But now I can feel when I need to use it*”
IP3	Low “*Because the medicine does not give you an immediate effect. You still feel bad even after [using] the medicine. Actually it takes a long time before the medicine works*”	High (for medicine) *[2nd author:* “*Where are you on a scale from 1–10 about remembering to use the medicine, where 1 is bad and 10 is good?*”*]* “*I actually think I am about 7–8*”	Low (for medicine) “*Sometimes I take it [medicine] twice a day. It depends on how I feel… I actually think [my asthma] has improved over time, therefore I am thinking about quitting [using medicine]*”
IP4	High for reliever, low for controller “*I don’t think I see a difference in the one [controller] that I use morning and evening. But more to a higher degree with the one I use when necessary; its effect I feel right away*”	High (for medicine) “*[I] just put it by my bed so that I remember to use it both morning and evening. And the blue one [reliever] I always have with me in the car*”	High (for medicine) “*And right during my workout, I felt I needed air, but I did not get enough /…/I took my medicine right away*”
IP5	High for reliever, low for controller: “*I use the blue [reliever] one [mostly], that is probably the strongest [medicine]*”	High (in general) “*I am probably very positive. I am always lively. I always have energy*”	High (for medicine) “*I had started coughing/…/so I went home and took my medicine and then I went back to the party*”
IP6	High “*[Terbutaline, reliever]. That is [used] when I feel worst/…/if I don’t use the brown one [controller] for a period, then there can come a time where I get an exacerbation and am hospitalised*”	Low (for medicine) “*I am irresponsible regarding my own medicine. But I always remember my mom’s*”	Low (for medicine) “*With asthma, it is first when I start to feel ill that I remember to use it. Once, two months passed where I didn’t use my medicine. Not one single [time]*”
IP7	Low “*If I use my medicine, I will probably get better, but I probably won’t be completely asthma-free*”	High (in general) “*I am very strong-willed and occasionally stubborn, but at the same time I see myself as a responsible, mature and sensible young woman*”	Low (for medicine) “*Would say I am pretty bad at using the one I need daily. So when I feel ill, I get motivated to use it*”
IP8	High “*Sometimes the two don’t help, the brown and the red [controllers]. Then, it is only the blue [reliever] that helps, because it works faster than the other two*”	High (for medicine) *[2nd author:* “*Where are you on a scale from 1–10 about remembering to use the medicine, where 1 is bad and 10 is good?]* “*Maybe 8*”	High (for medicine) “*I use my medicine morning and evening*”
IP9	Low “*I was told that it would go away when I used my inhaler daily for a while, but I haven’t seen the effect yet. When I don’t use it for 3–4 days, I get a severe cough and breathing difficulties*”	High (in general) “*I am very outgoing, helpful, and can be aggressive but also friendly*”	Low (for medicine) “*I don’t need to do anything [to remember the medicine]. When I don’t use it my cough reminds me right away*”
IP10	High “*I can really feel how the medicine helps*”	High (for medicine) “*I use the brown one [controller] morning and evening, and the blue [reliever] when needed. The blue I also use before exercising*”	High (for medicine) “*I set the alarm so I remember to use my medicine*”

## Supplementary Materials

The interview guides are available online at http://www.mdpi.com/2226-4787/6/3/73/s1, List S1: Individual and focus group interview guides.
