# Supplementary material for: Young Muslim Women Living with Asthma in Denmark: A Link between Religion and Self-Efficacy
Source: Pharmacy (Basel). 2018 Jul 23;6(3):73. doi: 10.3390/pharmacy6030073 (PMC6165087; doi:10.3390/pharmacy6030073)
Supplement: Supplementary file 1 [file pharmacy-06-00073-s001.pdf]

Interview guide – individual interviews

Welcome including information about anonymity, confidentiality and estimated duration of interview

Background

1. How old are you?
2. What do you do for a living?
3. What is your city of residence?
4. What is your country of origin?
5. How long have you been living in Denmark?
    a. If since birth, how long have your parents been living in Denmark?

Disease history

1. Can you please tell me how you or your parents discovered that you have asthma?
2. How long have you had asthma?
3. Do others in your family have asthma?

Compliance

1. What medicines do you use?
    a. How often?
2. Are you the one primarily in charge of your medication?
    a. Do your parents or others assist you in remembering to use your medicines?
3. How does the medicine help you – if it does?
    a. And why?
4. How do you take your medicine?
5. What did you do the last time you experienced an exacerbation?
6. How often do you get asthma exacerbations?
7. How much do your parents/others help you with your medication?
8. Do you experience problems with your medication?
9. Do you lack information about the effects or adverse effects of your medication?
10. Do you have any other diseases?
11. Do you always carry your medicine with you?
12. Do you remember to use your medicine on time and according to your doctor's instructions?
13. How do you remember to take your medicine?
14. How can anyone get used to taking medicine?
15. Do you buy medicine from your country of origin?
16. Do your parents/others buy medicine for you from countries other than Denmark?

Social status

1. Do you buy your medicines or do your parents/others buy it for you?
2. Have you or your parents at some point purposefully not bought the medicine?
    a. If yes, why?

Culture/ethnicity

1. Do you know if there is a difference between the healthcare system in Denmark and in your country of origin?
2. Can you describe the attitude towards doctors in your culture?
3. How accessible are pharmacies in your country of origin?

Religion

1. How do you think religion can help you manage your asthma?
2. How does your religion influence your medicine use?
3. Do you feel that you can pray, for example, if your disease is worsening?
4. Do you want to change anything about your medicine use during Ramadan?
    a. Will you fast, or what are your plans during Ramadan?

Circumstances due to asthma

1. Have you been absent from school due to asthma?
    a. If yes, how much does that affect your ability to complete your education?
2. Does asthma limit your physical activities?
3. Does asthma limit you in other ways?
4. Have you been hospitalised previously due to asthma?
    a. If yes, how often?
5. Do you know the consequences of not taking your medicine?
6. Have you previously been hospitalised because you forgot to take your medicine?

Self-efficacy

1. How do you experience life with a chronic disease?
2. Have you ever been in a situation where you needed to explain about your asthma to others?
3. How do you handle the situation when exacerbations occur?
4. Where do you get your inspiration and stamina? What is your source?
5. Do you use the Internet for information about medicine?
6. How would you describe your personality? (positive/negative)
7. What is your way forward to conquering your asthma?
8. When do you feel in control of your asthma?
9. Have you achieved what you want with your asthma?
10. What is your source of motivation in life?
11. How do you motivate yourself to have control over your asthma?

12. Can you control your asthma exacerbations?
13. If you were to give advice to other women with asthma, what would it be?

Gender

1. Do you think it is better to be a woman with asthma rather than a man with asthma?
2. Have you noticed a worsening of your asthma prior to or after your period?

Smoking

1. Do you smoke?
    a. If yes, how much?

Healthcare system

1. How was your last visit to your GP's office?
2. Did you get counselling about the use of asthma medicine there?
3. Have you previously participated in other studies about asthma?
4. Do you feel that your GP supports you in how to handle the disease?
5. What information did you receive at the pharmacy about your medicine?
6. What is your or your parent's view of the pharmacy?

Additional aspects

1. Would you like to add something?
2. Are there other aspects you want to ask me about?
3. Would you like to participate in a focus group interview with other women with asthma?

Interview guide – focus group interview

Welcome

Ramadan

1. How have you handled Ramadan and asthma?
2. Did you fast?
    a. If yes, did you experience any problems?
    b. Why did you start fasting now that, according to Islamic beliefs, you are exempt from fasting because you have a chronic disease?
3. How did you use your asthma medicine during Ramadan?
4. Do you have an agreement with your doctor about how to use your medicine during Ramadan?

5. Did you have exacerbations due to fasting either this Ramadan or in previous years?
6. What precautions are you taking relating to Ramadan and your medicine treatment?
7. From where did you receive information about Ramadan telling you that according to Islam you are allowed to take medicine during this month?
8. What should you prioritize most: fasting or medicine treatment?
9. How can age or gender be essential for an asthmatic in terms of fasting or not fasting during Ramadan?
10. Do your parents or others influence your decision to fast during Ramadan?

Ethnicity

1. What makes you young women of ethnicity other than Danish?
2. Do you think that there is a difference between young female asthma patients with ethnicity other than Danish compared to young, Danish female asthma patients?
3. In your view, what determines ethnic difference?
4. How do you experience that ethnicity can play a crucial role in health and treatment?
5. What ethnic group does each of you belong to?

Compliance

1. How do you use your medicines?
2. How can age be important for how one uses asthma medicine?
    a. And for taking responsibility for yourself and remembering your medicine on time?
